# Joint Effect of Urinary Total Arsenic Level and VEGF-A Genetic Polymorphisms on the Recurrence of Renal Cell Carcinoma

**DOI:** 10.1371/journal.pone.0145410

**Published:** 2015-12-23

**Authors:** Shu-Mei Yang, Chao-Yuan Huang, Horng-Sheng Shiue, Shu-Pin Huang, Yeong-Shiau Pu, Wei-Jen Chen, Ying-Chin Lin, Yu-Mei Hsueh

**Affiliations:** 1 School of Public Health, College of Public Health and Nutrition, Taipei Medical University, Taipei, Taiwan; 2 Department of Urology, National Taiwan University Hospital, College of Medicine National Taiwan University, Taipei, Taiwan; 3 Department of Chinese Medicine, Chang Gung Memorial Hospital and College of Medicine, Chang Gung University, Taoyuan, Taiwan; 4 Department of Urology, Kaohsiung Medical University Hospital, Kaohsiung, Taiwan; 5 Department of Urology, Faculty of Medicine, College of Medicine, Kaohsiung Medical University, Kaohsiung, Taiwan; 6 Department of Family Medicine, Shung Ho Hospital, Taipei Medical University, Taipei, Taiwan; 7 Department of Health Examination, Wan Fang Hospital, Taipei Medical University, Taipei, Taiwan; 8 Division of Family Medicine, School of Medicine, Taipei Medical University, Taipei, Taiwan; 9 Department of Public Health, School of Medicine, College of Medicine, Taipei Medical University, Taipei, Taiwan; University of Rajshahi, BANGLADESH

## Abstract

The results of our previous study suggested that high urinary total arsenic levels were associated with an increased risk of renal cell carcinoma (RCC). Germline genetic polymorphisms might also affect cancer risk and clinical outcomes. Vascular endothelial growth factor (VEGF) plays an important role in vasculogenesis and angiogenesis, but the combined effect of these factors on RCC remains unclear. In this study, we explored the association between the *VEGF-A* -2578C>A, -1498T>C, -1154G>A, -634G>C, and +936C>T gene polymorphisms and RCC. We also evaluated the combined effects of the *VEGF-A* haplotypes and urinary total arsenic levels on the prognosis of RCC. This case-control study was conducted with 191 RCC patients who were diagnosed with renal tumors on the basis of image-guided biopsy or surgical resections. An additional 376 age- and gender-matched controls were recruited. Concentrations of urinary arsenic species were determined by a high performance liquid chromatography-linked hydride generator and atomic absorption spectrometry. Genotyping was investigated using fluorescent-based TaqMan allelic discrimination. We observed no significant associations between *VEGF-A* haplotypes and RCC risk. However, the *VEGF-A* ACGG haplotype from *VEGF-A* -2578, -1498, -1154, and -634 was significantly associated with an increased recurrence of RCC (OR = 3.34, 95% CI = 1.03–10.91). Urinary total arsenic level was significantly associated with the risk of RCC in a dose-response manner, but it was not related to the recurrence of RCC. The combination of high urinary total arsenic level and *VEGF-A* risk haplotypes affected the OR of RCC recurrence in a dose-response manner. This is the first study to show that joint effect of high urinary total arsenic and *VEGF-A* risk haplotypes may influence the risk of RCC recurrence in humans who live in an area without obvious arsenic exposure.

## Introduction

Renal cell carcinoma (RCC) is the most common malignancy of the kidney and is one of the most common cancers in western countries [1]. In Taiwan, RCC is estimated to account for 0.9% of all cancers [2]. Previous studies have shown that environmental risk factors such as cigarette smoking and arsenic exposure increase the risk of RCC [3, 4]. Genetic variations in angiogenesis-related genes have also been reported to be involved in the etiology of RCC [5, 6].

Human exposure to arsenic via drinking water increases the risk of skin cancer, lung cancer, and urothelial carcinoma [7–9]. Several studies in Bangladesh and Taiwan examined the association between arsenic exposure in drinking water and RCC risk [10, 11], but a case-control study in Northern Chile reported no association [12]. Our previous study suggested that high urinary total arsenic levels were related to RCC, even among subjects living in an area without obvious arsenic exposure [4]. The mechanism for arsenic-induced carcinogenesis remains unclear.

Vascular endothelial growth factor (VEGF), which is well known as an essential factor for vascular permeability, plays an important role in vasculogenesis and angiogenesis [13]. The VEGF family comprises several different proteins, including VEGF-A, VEGF-B, VEGF-C, VEGF-D, VEGF-E, VEGF-F, and placental growth factor[14]. VEGF-A has been studied more than the other proteins of the VEGF family [15]. The *VEGF-A* gene is located on chromosome 6p21.3 and contains 8 exons [16]. Several single-nucleotide polymorphisms (SNPs) in the *VEGF* gene have been reported to affect gene expression [17]. Specifically, polymorphisms in the *VEGF-A* promoter region (-2578C>A [rs699947], -1498T>C [rs833061, also named as -460T/C], and -1154G>A [rs1570360]), 5’-untranslated region (-634G>C [rs2010963, also named as +405G/C]), and 3’-untranslated region (+936C>T [rs3025039]) have been associated with VEGF expression [17–19]. Study subjects with the *VEGF-A* -2578 A allele [20] and the -1498 CT and TT genotypes [21] had an increased risk of RCC, but a Spanish study showed that *VEGF-A* -2578 and -1498 polymorphisms did not impact RCC risk [22]. Previous studies reported that the -1154 G allele, the +936 CC genotype, and the +936C allele were associated with an increased risk of breast cancer [23, 24], but a study in France did not reveal any significant associations with RCC risk [21]. For the +405G>C polymorphism, the +405 C allele was associated with a decreased risk of gastric cancer [25], but this polymorphism in *VEGF-A* was not related to RCC risk [22].

The SNPs in *VEGF* have also been reported to be correlated with the progression and survival of RCC. A previous study reported that RCC patients who carried the -2578 AA genotype or the A allele had a higher tumor stage, a larger tumor size, and an increased risk of death than patients who carried the -2578 CC genotype or C allele; patients who carried the -634 CC genotype or -634 C allele also had a larger tumor size than patients who carried the GG genotype or G allele, respectively [26]. Scartozzi et al. found that RCC patients with -2578 CC [rs699947], -1498 TT [rs833061], and -634 CC [rs2010963] genotypes had worse progression-free survival and overall survival than patients with -2578 AA+AC, -1498 CC+CT, and -634 GG genotypes, respectively [27]. Kawai et al. reported that the -2578 CA+AA genotypes were associated with less frequent lymph node metastasis than the -2578 CC genotype and that the -1154 GA+AA genotypes and A allele were associated with smaller tumors and lower tumor stage than the GG genotype or G allele, respectively. The -634 polymorphism had no significant clinical effect on RCC progression or prognosis [28]. Kim et al. demonstrated that the combination of the +936 CC and *VEGFR2* 889 GG genotypes was associated with a poor prognosis of RCC compared to all other genotypes [29]. However, Saenz-Lopez et al. reported that the *VEGF-A* -2578, -1498, -634, and +936 polymorphisms were not correlated with clinicopathological characteristics or the prognosis of RCC [22], and Zhong et al. reported no association between the *VEGF-A* -1154 polymorphism and RCC progression [26]. Therefore, the associations between genetic variations in the *VEGF-A* gene and RCC remain controversial.

Recently, low levels of arsenic were reported to have stimulated VEGF expression in human uroepithelial cells [30, 31]. Also, urinary VEGF levels were reportedly increased in copper-smelting workers who were exposed to arsenic and these urinary VEGF levels were significantly associated with urinary arsenic levels [32]. Another study reported a dose-response relationship between the arsenic concentrations of water, hair, and nails and serum VEGF levels in a population with chronic arsenic exposure [33]. Several studies have reported that arsenic increases VEGF-A levels; however, the joint effect of urinary total arsenic levels and *VEGF-A* polymorphisms on RCC remains unclear. In this study, we explored the associations between gene polymorphisms of *VEGF-A* -2578C>A [rs699947], -1498T>C [rs833061], -1154G>A [rs1570360]), -634G>C [rs2010963]), and +936C>T [rs3025039] and RCC. We also evaluated the combined effects of these SNPs and urinary total arsenic level on the risk and prognosis of RCC.

## Material and Methods

### Study subjects

For this study, we recruited 191 patients who were diagnosed with RCC. Pathological verification of RCC was completed by image-guided biopsy or surgical resection of renal tumors. An additional 376 healthy control subjects were recruited. The control subjects had no evidence of RCC or any other malignancy and were matched with the cases on the basis of age (± 5 years) and gender. All study subjects were recruited from the Departments of Urology and from adult and senior citizen health examinations at National Taiwan University Hospital, Taipei Medical University Hospital, and Taipei Municipal Wan Fang Hospital between November 2006 and June 2009. All three hospitals were located within Taipei City, approximately 200 to 300 km from arsenic-contaminated areas in Taiwan; none of the RCC cases or controls came from the arsenic-contaminated areas. There is no specific industrial process in Taipei City that creates arsenic pollution. The majority of study participants (≥ 80%) lived in Taipei City and drank tap water supplied by the Taipei Water Department of the Taipei City Government. The average arsenic concentration of tap water in Taipei City is 0.7 μg/L, but concentrations range from non-detectable to 4.0 μg/L, which is lower than the standard guideline value set by the World Health Organization of 10 μg/L [34]. The Research Ethics Committee of National Taiwan University Hospital approved the study (approval reference number 9100201527), and all of the study subjects provided written informed consent before questionnaire interview and specimen collection. This study was undertaken in accordance with the World Medical Association Declaration of Helsinki.

Well-trained interviewers conducted face-to-face interviews of each study subject using a structured questionnaire. The questionnaire included demographic and socioeconomic characteristics, lifestyle habits such as cigarette smoking, consumption of alcohol, tea, and coffee, and disease histories of hypertension, diabetes, and urolithiasis [4]. According to the work history of the questionnaire, none of the participants were occupationally exposed to arsenic. Spot urine samples were collected at the time of enrollment and stored at -20°C until analysis of urinary arsenic species. At the same time, peripheral blood samples were collected in ethylene diamine tetra acetic acid (EDTA) vacuum syringes. The DNA was extracted and stored at -80°C until the measurement of gene polymorphisms.

### Determination of urinary arsenic species

Frozen urine samples were thawed at room temperature, dispersed using ultrasonication, and filtered through a Sep-Pak C18 column (Mallinckrodt 188 Baker, Phillipsburg, NJ, USA). A urine aliquot of 200 μL was used for the determination of arsenic species by high-performance liquid chromatography (Merck Hitachi, Tokyo, Japan) with columns obtained from Phenomenex (Nucleosil, Torrance, CA, USA). The concentrations of arsenite (As^III^), arsenate (As^V^), monomethylarsonic acid (MMA^V^), and dimethylarsinic acid (DMA^V^) were quantified by hydride generator-atomic absorption spectrometry (PerkinElmer, Waltham, MA, USA) [35]. The standard reference material SRM 2670 (National Institute of Standards and Technology, Gaithersburg, MD, USA), which contains 480 ± 100 μg/L inorganic arsenic, was used as a quality standard and analyzed along with the urine samples. The mean value of SRM 2670 was determined to be 507 ± 17 μg/L (n = 4). Recovery rates for As^III^, As^V^, MMA^V^, and DMA^V^ ranged from 93.8% to 102.2%, with detection limits of 0.02, 0.08, 0.05, and 0.07 μg/L, respectively. The sum of As^III^, As^V^, MMA^V^, and DMA^V^ was defined as urinary total arsenic. Arsenic methylation capacity indices, including inorganic arsenic percentage (InAs%), MMA percentage (MMA%), and DMA percentage (DMA%), were calculated by dividing the concentration of each arsenic species by the urinary total arsenic concentration. Moreover, arsenobetaine from seafood is less harmful, which cannot form hydrides and cannot be detected with HG-AAS [36, 37]. The urinary total arsenic concentration was adjusted for urinary creatinine (mg/dL) to account for variations in hydration status[38]. The most widely used method is creatinine adjustment, which involves dividing the analyte concentration (μg/L) by the creatinine concentration (g/L). Results are then reported as weight of analyte per gram of creatinine (μg/g creatinine). However, many previous studies have shown that urinary creatinine concentrations can be affected by age, gender [39], muscle mass [40], and kidney function [41], and have suggested that urinary creatinine should be included in multiple regression models as a separate independent variable [42]. Urinary creatinine was measured by colorimetric assay on the Roche Modular P800 instrument (Roche Inc., Mannheim, Germany).

### Genotyping of VEGF-A

DNA was extracted using proteinase K digestion after phenol and chloroform extraction. The allelic discrimination of *VEGF-A* SNPs genotyping were investigated using fluorescent-based TaqMan allelic discrimination (Applied Biosystems assay IDs: C_8311602_10 [rs699947], C_11400863_20 [rs833061], C_1647379_10 [rs1570360], C_8311614_10 [rs2010963], and C_16198794_10 [rs3025039]) (S1 File). Briefly, SNP amplification assays including 10 ng of sample DNA in 10 μL of reaction solution contained in 5 μL of 2X TaqMan Universal PCR Mix (Applied Biosystems) and 0.5 μL of 20X probe/ primer assay mix were performed according to the manufacturer’s instructions. PCR cycling was completed using an ABI Prism 7500HT sequence detection system. Thermal cycling was initiated with a first denaturation step of 95°C for 10 min, followed by 40 cycles of denaturation at 92°C for 15 s and annealing at 60°C for 1 min. A random 10% of the study samples were repeated for quality control, and the results showed 100% concordance.

### Statistical analysis

The distribution of the polymorphisms was assessed by the goodness-of-fit chi-square test in the non-RCC control group for Hardy-Weinberg equilibrium. Linkage disequilibrium (LD) was calculated using the Haploview software, version 4.2 (Broad Institute, Cambridge, MA, USA) and Lewontin D′ expressed the strength of the LD. Student’s t-test was used to analyze the difference in urinary total arsenic levels and arsenic profiles between RCC patients and controls, and recurrent cases and non-recurrent cases. Odds ratios (OR) and 95% confidence intervals (CI) were estimated by multivariate logistic regression models to determine the associations between urinary total arsenic, *VEGF-A* gene polymorphisms, and RCC. Cutoff points for continuous variables were the respective tertiles of the controls or non-recurrent cases. *VEGF-A* haplotype was estimated on the basis of the expectation-maximization algorithm using the SAS/Genetics module (SAS Institute, Cary, NC, USA). Significance tests for the linear trend among ORs across exposure strata were calculated by categorizing exposure variables and by treating ordinal variables as continuous variables. We used the relative excess risk due to interaction (RERI), synergy index (SI) and the respective 95% CIs to investigate the additive interactions of *VEGF-A* polymorphisms and urinary total arsenic level. For the joint effect analysis, the cutoff point for urinary total arsenic concentration (17 μg/L) was the median value of the non-recurrent cases. The two indexes were presented by Rothman and the detailed calculation method described by the previous study [43]. The RERI > 0 and SI >1 indicate the positive interaction. All statistical tests were two-sided with a significance level of 0.05. Statistical analyses were performed using Statistical Analysis Software version 9.3 (SAS Institute, Cary, NC, USA).

## Results

The total of 567 participants in the study included 191 RCC patients and 376 age- and gender-matched healthy controls. The mean ages of the cases and controls were 58.47 ± 0.96 years and 59.77 ± 0.63 years, respectively. Table 1 shows the demographics and characteristics of the cases, controls, recurrences and non-recurrences. After adjusting for gender and age, subjects with parental ethnicity of Mainland Chinese and other ethnicities had a significantly lower RCC risk than those with a parental ethnicity of Fukien Taiwanese. Cigarette smoking status was not related with the OR of RCC. Occasional alcohol drinking was inversely associated with the OR of RCC (OR 0.10, 95% CI 0.05–0.23). Consumption of tea or coffee significantly decreased the OR of RCC. Disease histories of hypertension, diabetes, or urolithiasis significantly increased the OR of RCC. RCC patients who consumed alcohol had a significantly higher risk of RCC recurrence (OR 3.72, 95% CI 1.36–10.18) than those who did not drink alcohol. Patients with stage III +IV (OR 11.61, 95% CI 4.13–32.68) or grade G3+G4 (OR 4.35, 95% CI 1.58–12.00) disease had a significantly increased risk of RCC recurrence than patients with stage I +II or grade G1+G2 disease, respectively.

**Table 1:** Demographics and clinical characteristics of the study participants.

Variables		RCC cases (n = 191) n (%)	Controls (n = 376) n (%)	Age-gender adjusted OR (95%CI)	RCC recurrence (n = 22) n (%)	Non-recurrence (n = 169) n (%)	Age-gender adjusted OR (95%CI)
Gender	Male	123 (64.40)	245 (65.16)	1.00	14 (63.64)	109 (64.50)	1.00
	Female	68 (35.60)	131 (34.84)	1.03 (0.71–1.48)	8 (36.36)	60 (35.50)	1.06 (0.42–2.68)
Educational level	Elementary school or below	41 (18.13)	68 (21.47)	1.00	8 (36.36)	33 (19.53)	1.00
	High school	69 (33.87)	127 (36.13)	0.84 (0.51–1.40)	8 (36.36)	61 (36.09)	0.49 (0.16–1.52)
	College or above	81 (48.00)	180 (42.41)	0.66 (0.39–1.09)	6 (27.27)	75 (44.38)	0.29 (0.08–1.01)^±^
Paternal ethnicity	Fukien Taiwanese	136 (71.20)	218 (57.98)	1.00	16 (72.73)	120 (71.01)	1.00
	Hakka Taiwanese	20 (10.47)	37 (9.84)	0.87 (0.48–1.56)	2 (9.09)	18 (10.65)	0.83 (0.18–3.91)
	Mainland Chinese and other ethnicities	35 (18.32)	121 (32.18)	0.47 (0.31–0.73)**	4 (18.18)	31(18.34)	0.97 (0.30–3.12)
Maternal ethnicity	Fukien Taiwanese	143 (74.87)	227 (60.37)	1.00	18 (81.82)	125 (73.96)	1.00
	Hakka Taiwanese	17 (8.90)	37 (9.84)	0.73 (0.40–1.34)	1 (4.55)	16 (9.47)	0.43 (0.05–3.47)
	Mainland Chinese and other ethnicities	31 (16.23)	112 (29.79)	0.50 (0.28–0.71)**	3 (13.64)	28 (16.57)	0.75 (0.21–2.72)
Cigarette smoking status	Never	128 (67.37)	258 (68.62)	1.00	14 (63.64)	114 (67.86)	1.00
	Yes	26 (13.68)	58 (15.43)	0.89 (0.51–1.55)	2 (9.09)	24 (14.29)	0.75 (0.14–4.00)
	Occasional	36 (18.95)	60 (15.96)	1.30 (0.78–2.15)	6 (27.27)	30 (17.86)	1.70 (0.56–5.20)
Alcohol consumption	Never	147 (76.96)	220 (58.51)	1.00	13 (59.09)	134 (79.29)	1.00
	Yes	37 (19.37)	60 (15.96)	0.85 (0.52–1.38)	9 (40.91)	28 (16.57)	3.72 (1.36–10.18)*
	Occasional	7 (3.66)	96 (25.53)	0.10 (0.05–0.23)**	0 (0.00)	7 (4.14)	-
Tea drinking	Never	104 (54.74)	132 (35.11)	1.00	10 (45.45)	94(55.95)	1.00
	Yes	80 (42.11)	159 (42.29)	0.63 (0.43–0.91)*	11 (50.00)	69(41.07)	1.58 (0.62–4.02)
	Occasional	6 (3.16)	85 (22.61)	0.09 (0.04–0.21)**	1 (4.55)	5(2.98)	1.95 (0.21–1.59)
Coffee drinking	Never	138 (72.63)	188 (50.00)	1.00	17 (77.27)	121(72.02)	1.00
	Yes	49 (25.79)	98 (26.06)	0.66 (0.44–1.00)*	5 (22.73)	44(26.19)	0.83 (0.29–2.41)
	Occasional	3 (1.58)	90 (23.94)	0.05 (0.02–0.15)**	0 (0.00)	3(1.79)	-
Hypertension	No	111 (58.12)	280 (74.67)	1.00	12 (54.55)	99 (58.58)	1.00
	Yes	80 (41.88)	95 (25.33)	2.51 (1.69–3.73)**	10 (45.45)	70 (41.42)	1.13 (0.44–2.95)
Diabetes	No	161 (84.29)	348 (92.55)	1.00	17 (77.27)	144 (85.21)	1.00
	Yes	30 (15.71)	28 (7.45)	2.45 (1.41–4.27)**	5 (22.73)	25 (14.79)	1.66 (0.56–4.96)
Urolithiasis	No	155 (81.58)	345 (91.76)	1.00	20 (90.91)	135 (80.36)	1.00
	Yes	35 (18.42)	31 (8.24)	2.55 (1.51–4.29)**	2 (9.09)	33 (19.64)	0.41 (0.09–1.86)
Stage	I+II				8 (40.00)	148 (88.10)	1.00
	III+ IV				12 (6.00)	20 (11.90)	11.61 (4.13–32.68)**
Grade	G1+G2				8 (44.44)	114 (77.03)	1.00
	G3+G4				10 (55.56)	34 (22.97)	4.35 (1.58–12.00)**

Educational level was unavailable in 1 control; cigarette smoking status was unavailable in 1 patient; tea drinking habits were unavailable in 1 patient; coffee drinking habits were unavailable in 1 patient; history of hypertension was unavailable in 1 patient; history of urolithiasis was unavailable in 1 patient; disease stage was unavailable in 3 patients; disease grade was unavailable in 25 patients.

^+^0.05<P<0.10

*P<0.05

**P<0.01.

Table 2 presents comparisons of urinary arsenic profiles and urinary total arsenic levels between RCC cases and controls, and RCC recurrence and non-recurrences. Urinary total arsenic levels of the patients (22.22 ± 1.81 μg/L) were similar to those of matched controls (22.64 ± 1.07 μg/L). No differences of mean values of urinary total arsenic levels were observed between recurrent cases (22.87 ± 6.30 μg/L) and non-recurrent cases (22.13 ± 1.89 μg/L). Urinary creatinine in RCC patients was significantly lower than in controls (82.72 ± 4.23 vs. 132.5 ± 4.91 mg/dL, P< 0.01), and there was no significant difference in urinary creatinine levels between recurrent cases (71.66 ± 11.87 mg/dL) and non-recurrent cases (84.15 ± 4.52 mg/dL). After multivariate including creatinine adjustment, a dose-response relationship was evident in the significant association between urinary total arsenic level and the risk of RCC (P_trend_<0.05). According to the trend analysis of tertiles of exposure strata, urinary MMA% was negatively associated with the OR of RCC. This was in contrast to our previous study, which found that inefficient arsenic methylation capacity (i.e., high MMA^V^% and low DMA^V^%) was related to urothelial carcinoma [9]. At present, we cannot explain the reason for this difference. In contrast, arsenic methylation capacity indices and urinary total arsenic level were not significantly different between patients with a clinical recurrence and those without a recurrence.

**Table 2:** Odds ratios of RCC and RCC recurrence for urinary arsenic profiles in the study participants.

Variables	RCC cases n (%)	Controls n (%)	Multivariate adjusted OR^a^ (95%CI)	Variables	RCC recurrence n (%)	Non-recurrence n (%)	Multivariate adjusted OR^c^ (95%CI)
InAs (%)				InAs (%)			
≤2.38	67 (35.08)	126 (33.51)	1.00	≤2.39	11 (50.00)	57 (33.73)	1.00
2.38–5.67	49 (25.65)	126 (33.24)	0.79 (0.47–1.33)	2.39–7.23	4 (18.18)	56 (33.14)	0.39 (0.10–1.52)
>5.67	75 (39.27)	125 (33.24)	1.38 (0.85–2.24)	>7.23	7 (31.82)	56 (33.14)	0.28 (0.08–1.05)^±^
MMA (%)				MMA (%)			
≤2.404	87(45.55)	126 (33.51)	1.00^#^	≤1.36	6 (27.27)	57 (33.73)	1.00
2.404–7.87	58 (30.37)	125 (33.24)	0.68 (0.42–1.12)	1.36–4.92	7 (31.82)	56 (33.14)	0.63 (0.16–2.47)
>7.87	46 (24.08)	125 (33.24)	0.43 (0.26–0.73)**	>4.92	9 (40.91)	56 (33.14)	0.70 (0.19–2.53)
DMA (%)				DMA (%)			
≤85.25	58 (30.37)	126 (33.51)	1.00	≤86.88	9 (40.91)	57 (33.73)	1.00
85.25–93.37	51 (26.70)	125 (33.24)	1.05 (0.62–1.78)	86.88–94.84	7 (31.82)	56 (33.14)	1.03 (0.29–3.62)
>93.37	82 (42.93)	125(33.24)	1.60 (0.98–2.62)^±^	>94.84	6 (27.27)	56 (33.14)	1.30 (0.35–4.81)
Urinary total arsenic (μg/L)				Urinary total arsenic (μg/L)			
≤10.52	66 (34.55)	126 (33.51)	1.00^#^ ^,^ ^b^	≤10.47	9 (40.91)	57 (33.73)	1.00^d^
10.52–24.23	66 (34.55)	125 (33.24)	1.72 (0.96–3.08)^±^	10.47–22.65	5(22.73)	56 (33.14)	0.74 (0.18–3.10)
>24.23	59 (30.89)	125 (33.24)	4.07 (2.02–8.19)**	>22.65	8 (36.36)	56 (33.14)	1.94 (0.35–10.80)

Total arsenic = As^III^+As^V^+MMA^V^+DMA^V^.

InAs% = (As^III^+As^V^)/total arsenic×100%.

MMA% = MMA^V^/total arsenic×100%.

DMA% = DMA^V^/total arsenic×100%.

^a^ Adjusted for age, gender, parental ethnicity, alcohol consumption, tea drinking, coffee drinking, and histories of hypertension, diabetes, and urolithiasis.

^b^ Adjusted for age, gender, parental ethnicity, alcohol consumption, tea drinking, coffee drinking, and histories of hypertension, diabetes, urolithiasis, and creatinine.

^c^ Adjusted for age, gender, alcohol consumption, and disease stage.

^d^ Adjusted for age, gender, alcohol consumption, disease stage, and creatinine.

^+^0.05<P<0.10,

**P<0.01.

^#^ P< 0.05 for trend test

**Fig 1:**
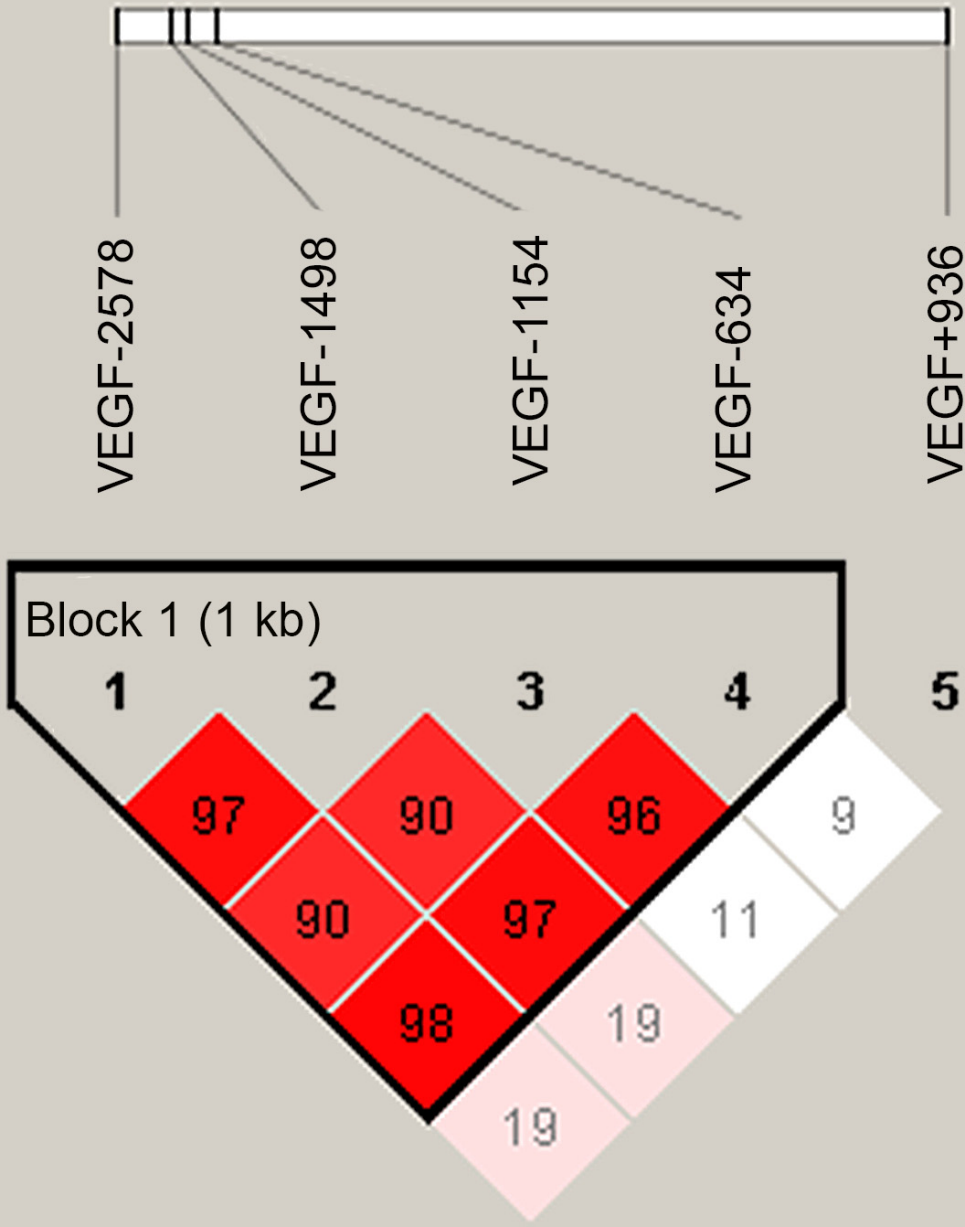
Linkage disequilibrium (LD) plot of five *VEGF-A* SNPs in all subjects. The Lewontin D' values among the SNPs investigated are shown.

Table 3 presents comparisons of *VEGF-A* genotypes and haplotypes between RCC cases and controls, and recurrence and non-recurrence. Similar frequencies were found in the distribution of *VEGF-A* -2578C>A, -1498T>C, -1154G>A, -634G>C, and +936C>T polymorphisms between RCC patients and healthy controls. The Lewontin D′ values of *VEGF-A* -2578, -1498, -1154, and -634 were all > 0.90, while the +936 polymorphism had a low Lewontin D′ (Fig 1). Haplotype frequencies of less than 5% were excluded from the RCC risk analysis. Four combinations in the haplotype analysis of *VEGF-A* -2578, -1498, -1154, and -634 are shown in Table 3. No significant associations were observed between any of the *VEGF-A* haplotypes and RCC risk. However, a significant association was found between the *VEGF-A* haplotype and clinical recurrence. RCC patients who carried haplotype ACGG had a significantly higher OR of RCC recurrence than patients with the low-risk haplotype CTGC (3.34, 95% CI 1.03–10.91).

**Table 3:** Distribution of *VEGF-A* genotypes and haplotypes in the study participants.

Variables		RCC cases n (%)	Controls n (%)	Age-gender adjusted OR (95%CI)	Multivariate adjusted OR^c^ (95%CI)	RCC recurrence n (%)	Non-recurrence n (%)	Age-gender adjusted OR (95%CI)	Multivariate adjusted OR^d^ (95%CI)
*VEGF-A* -2578 C>A	C/C	106 (55.50)	200 (53.19)	1.00	1.00	13 (59.09)	93 (55.03)	1.00	1.00
	C/A	75 (39.27)	153 (40.69)	0.94 (0.65–1.35)	1.00 (0.66–1.54)	6 (27.27)	69 (40.83)	0.62 (0.22–1.71)	0.66 (0.20–2.17)
	A/A	10 (5.24)	23 (6.12)	0.83 (0.38–1.81)	0.74 (0.30–1.82)	3 (13.64)	7 (4.14)	3.01 (0.69–13.18)	3.91 (0.66–23.19)
*VEGF-A* -1498 T>C	T/T	106 (55.50)	201 (53.46)	1.00	1.00	13 (59.09)	93 (55.03)	1.00	1.00
	T/C	76 (39.79)	150 (39.89)	0.97 (0.68–1.40)	1.06 (0.69–1.62)	6 (27.27)	70 (41.42)	0.61 (0.22–1.69)	0.66 (0.20–2.16)
	C/C	9 (4.71)	25 (6.65)	0.71 (0.32–1.57)	0.73 (0.30–1.81)	3 (13.64)	6 (3.55)	3.52 (0.78–15.89)	4.56 (0.73–28.59)
*VEGF-A* -1154 G>A	G/G	131 (68.59)	256 (68.09)	1.00	1.00	19 (86.36)	112 (66.27)	1.00	1.00
	G/A	52 (27.23)	99 (26.33)	1.04 (0.70–1.55)	1.10 (0.69–1.75)	1 (4.55)	51 (30.18)	0.11 (0.01–0.85)*	0.11 (0.01–0.93)*
	A/A	8 (4.19)	21 (5.59)	0.75 (0.32–1.74)	0.96 (0.35–2.66)	2 (9.09)	6 (3.55)	1.99 (0.37–10.64)	3.14 (0.48–20.74)
*VEGF-A* -634 G>C	G/G	62 (32.46)	136 (36.17)	1.00	1.00	7 (31.82)	55 (32.54)	1.00	1.00
	G/C	90 (47.12)	173 (46.01)	1.14 (0.77–1.70)	1.20 (0.76–1.90)	11 (50.00)	79 (46.75)	1.09 (0.39–3.00)	0.92 (0.29–2.95)
	C/C	39 (20.42)	67 (17.82)	1.26 (0.77–2.07)	1.22 (0.69–2.17)	4 (18.18)	35 (20.71)	0.91 (0.25–3.36)	0.50 (0.09–2.88)
*VEGF-A* +936 C>T^a^	C/C	122 (63.87)	232 (61.87)	1.00	1.00	15 (68.18)	107 (63.31)	1.00	1.00
	C/T	59 (30.89)	121 (32.27)	0.91 (0.62–1.34)	0.98 (0.62–1.53)	6 (27.27)	53 (31.36)	0.82 (0.30–2.26)	0.95 (0.30–3.00)
	T/T	10 (5.24)	22 (5.87)	0.85 (0.38–1.83)	0.72 (0.29–1.78)	1 (4.55)	9 (5.33)	0.79 (0.09–6.68)	0.75 (0.07–7.74)
		frequency	frequency			frequency	frequency		
*VEGF-A* haplotype^b^	CTGC	0.440	0.402	1.00	1.00	0.432	0.441	1.00	1.00
	CTGG	0.301	0.317	0.87 (0.65–1.16)	0.87 (0.62–1.23)	0.273	0.305	0.91 (0.42–1.95)	1.16 (0.48–2.79)
	ACAG	0.165	0.173	0.88 (0.62–1.26)	0.99 (0.66–1.50)	0.091	0.175	0.52 (0.17–1.61)	0.72 (0.21–2.47)
	ACGG	0.079	0.086	0.85 (0.53–1.36)	0.78 (0.45–1.33)	0.182	0.065	2.88 (1.12–7.42)*	3.34 (1.03–10.91)*

^a^ *VEGF-A* +936 C/T, information was unavailable for 1 patient.

^b^ *VEGF-A* haplotype (-2578)-(-1498)-(-1154)-(-634); haplotype frequencies of < 5% were excluded from the haplotype analysis.

^c^ Adjusted for age, gender, parental ethnicity, alcohol consumption, tea drinking, coffee drinking, and histories of hypertension, diabetes, and urolithiasis.

^d^ Adjusted for age, gender, alcohol consumption, and disease stage.

*P<0.05

As shown in Table 4, we further analyzed the combination of urinary total arsenic and *VEGF-A* haplotypes on the risk of RCC recurrence after adjusting for other confounding factors. Compared with RCC patients who carried *VEGF-A* CTGC, CTGG, and ACAG haplotypes and had low urinary total arsenic (≤ 17 μg/L), patients who had high urinary total arsenic (>17 μg/L) and carried the *VEGF-A* ACGG haplotype had a significantly increased OR of recurrence of 7.21 (95% CI 1.61–32.31) after multivariate adjustment (P_trend_ = 0.02). Additive interaction was found between *VEGF-A* haplotype and urinary total arsenic level (RERI = 5.36, 95% CI -5.00–15.73; SI = 7.38, 95% CI 0.16–332.33).

**Table 4:** The joint effects of urinary total arsenic and *VEGF-A* haplotypes on the risk of RCC recurrence.

*VEGF-A* haplotypes^a^	Urinary total arsenic	RCC recurrence Frequency	Non-recurrence Frequency	Multivariate adjusted OR^b^ (95%CI)
CTGC+CTGG+ACAG	≤17μg/L	0.488	0.472	1.00
	>17μg/L	0.326	0.463	1.44 (0.50–4.12)
ACGG	≤17μg/L	0.047	0.030	1.40 (0.20–9.95)
	>17μg/L	0.140	0.036	7.21 (1.61–32.31)**
				P_trend_ = 0.02
RERI				5.36 (-5.00–15.73)
Synergy index				7.38 (0.16–332.33)

^a^ *VEGF-A* haplotype (-2578)-(-1498)-(-1154)-(-634); haplotype frequencies of < 5% were excluded from the haplotype analysis.

^b^ Adjusted for age, gender, alcohol consumption, disease stage, and creatinine.

**P<0.01.

## Discussion

In the present study, we observed no significant differences in *VEGF-A* genotype frequencies between RCC cases and healthy controls. However, the *VEGF-A* ACGG haplotype was significantly associated with the clinical recurrence of RCC. Additionally, urinary total arsenic level was significantly associated with the OR of RCC, which was similar to the findings of our previous study [44]. Further, a significant dose-response relationship was observed in the joint effect of the *VEGF-A* ACGG haplotype and high urinary total arsenic on the risk of RCC recurrence.

Several reports have been published regarding the association of *VEGF-A* polymorphisms and various types of cancer, including prostate, renal, and lung cancers [21, 45, 46]. Ajaz et al. reported that *VEGF-A* -2578 CA+AA genotypes and the A allele were significantly associated with risk of RCC [20]. However, our current study indicated that neither the *VEGF-A* -2578, -1498, -1154, -634, and +936 polymorphisms nor the haplotypes of -2578, -1498, -1154, and -634 modified the risk of RCC. These findings are similar to the findings of a study conducted in Spanish patients [22]. Further, Jang et al. did not observe an association between the *VEGF-A* -2578C>A polymorphism and the development of colorectal cancer [47], and Wang et al. reported that the -1498T>C polymorphism was not associated with urinary tract urothelial carcinoma or bladder cancer in a Taiwanese population [48]. A case-control study in a Chinese population has suggested that the *VEGF* -1498 CC genotype was significantly increased risk of RCC [49] and Bruyere et al. reported that patients who carried *VEGF-A* -1498 CT or TT genotypes had an increased risk of developing cancer [21], which might be due to the fact that the *VEGF* -1498 polymorphisms were not in Hardy-Weinberg equilibrium. Moreover, Bruyere et al. also reported that the *VEGF-A* -1154, 1634, and +936 polymorphisms were not associated with RCC risk. A subsequent meta-analysis reviewed RCC data from five case-control studies; one of the studies similarly reported that the *VEGF-A* -1498T>C, -1154G>A, -634G>C, and +936C>T polymorphisms were not associated with the risk of RCC [50]. The percentages of the *VEGF*-*A* -1498 TT, TC, CC, +936 CC, CT, and TT genotypes among controls in our study were 53.46%, 39.89%, 6.65%, 61.87%, 32.27%, and 5.87%, respectively, which were similar to a study in the Taiwan population that reported percentages of 55.9%, 36.1%, 8.0%, 63.4%, 32.4%, and 4.2%, respectively [48]. In this study, we did not observe a significant difference in *VEGF-A* genotypes between RCC cases and controls. This may suggest that the *VEGF-A* polymorphisms are not associated with RCC, which is similar to a report about the lack of association of *VEGF-A* polymorphisms with bladder cancer [48].

Tumor characteristics such as large tumor size, tumor stage, and stage group are important factors for disease recurrence [51]. VEGF is a vascular permeability factor that mediates angiogenesis, endothelial cell growth, and vascular permeability [13]. A previous study showed that serum VEGF levels are higher in patients with recurrence than in patients without recurrence, and VEGF expression in RCC patients evaluated by immunohistochemistry was significantly correlated with a poor recurrence-free survival rate [52]. Kjaer-Frifeldt et al. reported that the haplotype combinations of CTC, ACG and ACG, ACG of three *VEGF* biallelic polymorphisms (-2578C>A, -1498C>T, and -634G>C) were significantly associated with a recurrence of colon cancer within 5 years [53], which is similar to the prognosis of RCC in our study. This finding also agrees with the findings of Howell et al. who reported that the *VEGF-A* -2578, -1154, -634 CAC haplotype was associated with less advanced cutaneous malignant melanoma [54]. The *VEGF-A* variant -2578 A allele was significantly associated with an increased risk of clinical recurrence of prostate cancer and chronic myeloid leukemia [55, 56]. A study conducted in Japan reported that the *VEGF-A* -2578 CC genotypes were correlated with poor survival in RCC patients compared with -2578 CA or CA+AA genotypes and that the -1154 GG genotype was associated with larger tumors, higher tumor stage, and higher stage grouping than the -1154 GA+AA genotype [28]. Moreover, the -1498 CC and -634 GG polymorphisms have been associated with poor disease-free survival and poor overall survival, respectively, for breast cancer [57]. Koukourakis et al. reported that the -2578CA genotype was correlated with higher VEGF levels than the CC genotype, but Shahbazi et al. reported that the -2578 CC genotype was associated with higher VEGF expression than the -2578 AA genotype [58, 59]. Thus, the reasons for different expression may depend on carcinoma type or ethnicity. In this study, none of the *VEGF* polymorphisms significantly influenced the risk of RCC recurrence. However, our analysis showed that the *VEGF-A* ACGG haplotype from -2578, -1498, -1154, and -634 was significantly associated with an increased risk of recurrence in RCC patients (OR = 3.34, 95% CI = 1.03–10.91). This suggests that high VEGF expression may be related to the recurrence of RCC. A previous study analyzed three *VEGF-A* haplotypes in MCF7 breast cancer cells by using a luciferase reporter assay that contained seven SNPs in the *VEGF-A* promoter. The study found that the ACGG haplotype from -2578 (also named -1540), -1498, -1154 (also named -116), and -634 showed higher promoter activity than ACAG and CTGC haplotypes [60]. Likewise, high VEGF expression has already been associated with a recurrence of non-muscle invasive bladder cancer [61–64].

The research carried out by Wang et al. showed that urinary levels of VEGF were increased in copper-smelting workers who were exposed arsenic [32]. Additionally, Rahman et al. reported that serum VEGF levels were increased with increasing total arsenic concentrations in drinking water, hair, and nails in Bangladesh [33]. Furthermore, low arsenic exposure have been associated with the progression of human cervical cancer cells and increased protein VEGF-A expression in NOD/SCID mice [65]. A previous study showed that arsenite-induced cell permeability increased through a reactive oxygen species (ROS)-VEGF pathway in mouse brain microvascular endothelial cells [66]. In a study of a human kidney cell line, the cytotoxic mechanisms of arsenic trioxide involved ROS production [67]. Evidence has shown that ROS are generated by low concentrations of arsenic and ROS increases the expression of VEGF in human uroepithelial cells. The expression of VEGF is partially mediated by mitogen-activated protein kinase (MAPK) and PI3K/AKT signaling pathways [31]. The MAPK signaling pathways play important roles in cell proliferation and differentiation. Additionally, in vivo models have shown that suppression of MAPK signaling pathways may inhibit renal cell carcinoma growth and tumor angiogenesis [68], which suggests that the MAPK pathway may play a role in RCC angiogenesis. Further, an in vitro study reported that arsenite increased levels of VEGF in human uroepithelial cells, and VEGF proteins in patients with urothelial carcinoma who lived in arsenic-contaminated areas were significantly higher than those of patients who lived in non-arsenic-contaminated areas [30]. Our study found that subjects living in an area without obvious arsenic exposure who had *VEGF-A* risk haplotypes and high urinary total arsenic had an increased risk of RCC recurrence in a dose-response manner. Taken together, high urinary total arsenic and risk haplotypes of *VEGF-A* may affect RCC recurrence, but this finding needs further investigation.

One of the limitations of this study is that urinary arsenic levels were evaluated with a single spot urine test. However, a previous long-term, prospective cohort study found that people exposed to low-to-moderate levels of inorganic arsenic from drinking water had constant urinary arsenic profiles, which suggests that a single determination of urinary arsenic and its species provides a useful biomarker of ongoing arsenic exposure and of individual arsenic metabolism [69]. Lacking of BMI and dietary habits information, as well as the arsenic levels of both hair and nails, was another limitation of the present study. In addition, some subjects with preexisting disease stop smoking and cause the no association between smoking and RCC may have caused the “sick quitter bias” [70].The sample size of this study was small and, as such, significant findings should be interpreted cautiously. This study was a case-control study; we cannot exclude the possibility that the association between urinary total arsenic levels and RCC might be the result of RCC and not the cause of RCC. Also, we did not measure either serum VEGF levels in patients or VEGF expression in tumor cells directly. Thus, the generalizability of our results is limited.

In conclusion, our study showed that the combination of high urinary total arsenic level and *VEGF-A* risk haplotypes affects the risk of RCC recurrence in a dose-response manner. To our knowledge, this is the first report to show that high urinary total arsenic combined with *VEGF-A* risk haplotypes may influence the risk of RCC recurrence in humans who live in an area without obvious arsenic exposure.

## Supporting Information

S1 File *VEGF-A* SNPs context sequences. (DOCX)
