# Supplementary material for: Joint Effect of Urinary Total Arsenic Level and VEGF-A Genetic Polymorphisms on the Recurrence of Renal Cell Carcinoma
Source: PLoS One. 2015 Dec 23;10(12):e0145410. doi: 10.1371/journal.pone.0145410 (PMC4689502; doi:10.1371/journal.pone.0145410)
Supplement: S1 File — (DOCX) [file pone.0145410.s001.docx]

**S1 Table. *VEGF-A* SNPs context sequences.**

| **Identified SNP** | **TaqMan assays** | **Context sequence [SNP]** |
| --- | --- | --- |
| rs699947 | C_8311602_10 | GCCAGCTGTAGGCCAGACCCTGGCA**[A/C]**GATCTGGGTGGATAATCAGACTGAC |
| rs833061 | C_11400863_20 | AGAGGGCTCAGCCTAATGGGATCTC**[C/T]**CCTCCCTTCCCTGGTTTGCATTCCT |
| rs1570360 | C_1647379_10 | AGCCCGGGCCCGAGCCGCGTGTGGA**[A/G]**GGGCTGAGGCTCGCCTGTCCCCGCC |
| rs2010963 | C_8311614_10 | CGCGCGGGCGTGCGAGCAGCGAAAG**[C/G]**GACAGGGGCAAAGTGAGTGACCTGC |
| rs3025039 | C_16198794_10 | GCATTCCCGGGCGGGTGACCCAGCA**[C/T]**GGTCCCTCTTGGAATTGGATTCGCC |

The forward and reverse primer sequences for PCR remain the intellectual property of the Applied Biosystems, and it cannot be revealed to the public.
